# *Eurosurveillance* reviewers in 2024

**DOI:** 10.2807/1560-7917.ES.2025.30.1.2501097

**Published:** 2025-01-09

**Authors:** Eurosurveillance editorial team

**Affiliations:** 1 European Centre for Disease Prevention and Control (ECDC), Stockholm, Sweden

**Keywords:** peer-review

The editorial team is grateful to all the experts who dedicated their time to peer-review our articles over the past 12 months. We wish to thank them and all those whose names are not listed below but gave helpful advice and support. We apologise to any reviewers whose names we may have missed.

Our reviewers in 2024 were:

Preben Aavitsland; Muna Abu Sin; Amos Adler; Philipp Agyeman; Josephine Aho; Tuomas Aivelo; Afsar Ali; Sheikh Taslim Ali; Kasim Allel; Vanessa Allen; Maria Joao Alves; Carmen Amaro; Antonella Amendola; Paul Anantharajah Tambyah; Nick Andrews; Jurica Arapovic; Takeshi Arashiro; Mardjan Arvand; Diane Ashiru-Oredope; Olle Aspevall; Zein Assad; Ana Avellon; Daniel Bailey; Heather Bailey; Craig Baker-Austin; Tamas Bakonyi; Vincenzo Baldo; Tala Ballouz; Christina Bancej; Mathieu Bangert; Nicolas Banholzer; José Barbas del Buey; Cyril Barbezange; Manoel Barral-Netto; Luisa Barzon; Stefano Bassetti; Nathalie Bastien; Ulrike Baum; Julien Beauté; Michael Beeton; Greta Bellinzona; Ronen Ben-Ami; Ria Benko; Kimberley Benschop; Beatrice Bercot; Matthijs Berends; Darlene Bhavnani; Silvia Bino; Carina Blackmore; Matteo Boattini; Daniela Boccolini; Pierre Bogaerts; Lucia Bonadonna; Marc Bonten; Wegene Borena; Andy Borman; Carles Borrego; Jordi Borrell Pique; Paolo Bosetti; Valérie Bouchez; Virginie Boulanger; Melissa Brady; Toon Braeye; Marieta Braks; Carina Brehony; Viviane Bremer; Els Broens; Colin Brown; Kevin Brown; Irene Burckhardt; Johanita Burger; Maria Bygdell; Sindy Böttcher; Håkon Bøås; Adriana Cabal Rosel; Saverio Caini; Caterina Candela; Jose Canevari; Michelle Canning; Bin Cao; Jun Cao; AnnaSara Carnahan; Jordi Casabona; Jean-Sebastien Casalegno; Caitlin A. Cassidy; Concetta Castilletti; Carlos Castillo-Salgado; Boudewijn Catry; Maiken Cavling Arendrup; Orlando Cenciarelli; Rachel Chalmers; Karen Champenois; Periklis Charalampous; André Charlett; Remi Charrel; Hualan Chen; Chiara Chiapponi; Cheng-Hsun Chiu; Eric Chow; Eirini Christaki; Peer Brehm Christensen; Jessie Chung; Daniela Cirillo; Jose Miguel Cisneros; Stephen Clark; Lucie Collineau; Marco Coppi; Teresa Coque; Joel Coste; Suzanne Cotter; Benjamin Cowling; Christiane Cuny; Fortunato D'Ancona; Ron Dagan; Francesca Dagostin; Viktor Dahl; Frederic Dalle; Eric Dannaoui; Raymund Dantes; Nicolas Dauby; Marieke de Cock; Sabine de Greeff; Genevieve Deceuninck; Francesco Defilippo; Nicolai Denzin; Jean-Claude Desenclos; Aditi Dey; Antonino Di Caro; Vincenzo Di Pilato; Maureen Diaz; Liselotte Diaz Högberg; Vitomir Djokic; Lisa Domegan; Florence Doucet-Populaire; Steven Drews; Christian Drosten; Rikard Dryselius; Ann-Sofi Duberg; Mariette F. Ducatez; Erwin Duizer; Robert Dyrdak; Paula Lucia Díaz Guevara; Javier Díez de los Ríos; Ralf Dürrwald; Tim Eckmanns; Michael Edelstein; Ulrich Elling; Hanne-Dorthe Emborg; Cécile Emeraud; Corinna Ernst; Helen Esser; Vicente Estrada; Steen Ethelberg; Helen Everett; Mirko Faber; Massimiliano Fabricci; Andrea Farnham; Paddy Farrington; Loïc Favennec; Maria Dolores Fernandez-Garcia; Laura Fernàndez-López; Nicolás Francisco Fernández Martínez; Cristina Ferreira; Julie Figoni; Thea Fischer; Joe Flannagan; Brendan Flannery; Nicole Forbes; James Ford; Diletta Fornasiero; Even Fossum; Leticia Franco; Christina Frank; Jamie Frankis; Eelco Franz; Rachel Freeman; Luca Freschi; Caroline Frey; Ingrid Friesema; Niels Frimodt-Møller; Aaron M. Frutos; Silvia Funke; Tuija Gadd; Juan Carlos Galan; Miguel Garcia-Deltoro; José-María García-Carrasco; Rebecca Garten Kondor; Rashi Gautam; Tatjana Gazibara; Davide Gentili; Antoine Gessain; Helge Giese; Nicolas Gilbert; Nicolo Girometti; Christian Giske; Federico Gobbi; Pere Godoy; Jessie J Goldsmith; Delia Goletti; Daniel Golparian; Cheng Gong; Ana Cristina Gonzalez-Perez; Prabhu Gounder; Manfred Green; Alexander L Greninger; Tamar Grossman; Thomas Grönthal; Peng Guan; Laura Guerrero-Latorre; Jesus Guinea; Vithiagaran Gunalan; Jean-Paul Guthmann; David L. Hahn; Ian Hall; Alvin X Han; Jörg Hans; Thomas Harder; Guy Harling; Ross Harris; Heli Harvala; Joana Haussig; Tetsuya Hayashi; Qiushui He; Florian Heger; Bernadette Hensen; Daniel Herrera-Esposito; Christian Herzog; David Higgins; Paul Higgins; Chelsea Himsworth; Vivian Hope; Win Htike; Angus Hughes; Ralph Huits; Ana Hurtado; Susanne Hyllestad; Victoria Indenbaum; Miren Iturriza-Gómara; Rossitza Ivanova Kurdova-Mintcheva; Radoslaw Izdebski; Jacques Izopet; Stéphanie Jacquinet; Irena Jakopanec; Andreas Jansen; Claire Jenkins; Cecilia Jernberg; Silvia Jimenez-Jorge; Kari Johansen; Michael Johansson; Nicholas Johnson; Vita Jongen; Femke Jongenotter; Carlijn Jordans; Annette Jurke; Sarah Kabbani; Ariel Karlinsky; Mark Katz; Sarah Kidd; Mi-Na Kim; Jason Kindrachuk; Pete Kinross; Robert Kirkcaldy; Lene Jung Kjær; Jonas Klingström; Don Klinkenberg; Ruth Klösch; Anke Kohlenberg; Philipp Kohler; Lisa Kohnle; Dimitra-Maria Koukou; Manuel Krone; Heinke Kunst; Oliver Kurzai; Kin On Kwok; Jeff Kwong; Sharon Kühlmann-Berenzon; Ida Laake; Katrien Lagrou; Margaret Lam; Theresa Lamagni; Mattijs Lambooij; Sylvie Larrat; Cristian Launes; Francois Lebreton; Sangnim Lee; Matti Lehtinen; Agnes Lepoutre; You Li; Giedrius Likatavicius; Troels Lillebaek; Chuan Kok Lim; Bruno Lina; Diane Lindsay; Adrian Lison; Mengyun Liu; Nicola Love; Theodore Lytras; Tinja Lääveri; Emma Löf; Sonja Löfmark Behrendtz; Miriam Maas; Liane Macdonald; Kimberly Mace; Raina MacIntyre; Shelley S. Magill; Carmelo Maida; Rui Malheiro; Alessandro Mancon; Sierk D Marbus; Matteo Marcantonio; Ellyn Marder; Gaetano Marrone; Katherine E. Marshall; Vito Martella; Melanie Marti; Josefa Masa-Calles; Sébastien Matamoros; Alberto Mateo Urdiales; Max Maurin; Stuart McLennan; Anika Meinen; Angeliki Melidou; Ella Mendelson; Lore Merdrignac; Stefano Merler; Marianna Meschiari; Kevin Messacar; Janey Messina; Devon S Metcalf; Patrick M. Meyer Sauteur; Jesus Mingorance; Maria Miragaia; Amir Mohareb; Marijke Molenaar-de Backer; Sara Monteiro Pires; Arnold Monto; Emanuele Montomoli; Orna Mor; Zohar Mor; Jacob Moran-Gilad; Alain Moren; Vanessa Morton; Joël Mossong; Aikaterini Mougkou; Laurent Moulin; Judith Mueller; Miguel Mulders; Arno Muller; Charlotte Munkstrup; David Muscatello; Otilia Mårdh; Maya Nadimpalli; Allen Nazareno; Richard Neher; Noele P Nelson; Julia Neufeind; Jens Nielsen; Georgios Nikolopoulos; Ran Nir-Paz; Hiroyuki Noda; Harold Noel; Hanna Nohynek; Yi Nong; Anne Christine Nordholm; Susan L. Norris; Norbert Nowotny; Baltazar Nunes; Dennis Nurjadi; Andrea Nwosu; Max R O'Donnell; Sean O'Leary; Doris Oberle; Eric Odhiambo Ochomo; Akihiro Ohkado; Andrea Olea; Monica Oleastro; Sonja Olsen; Natasa Opavski; Hana Orlíková; Hans Martin Orth; Richard Osei-Yeboah; Masaki Ota; Romain Palich; Marcus Panning; Katherine Paphitis; Dimitrios Paraskevis; Maria Pardos de la Gandara; Miguel Paredes; Elena Pariani; Daniel Parker; Michael D Parkins; Oana Pastiu; Robert Paton; Kishor Kumar Paul; Shlomit Paz; Malik Peiris; Tatjana Pekmezovic; André Peralta-Santos; Andreas Petersen; Patrizio Pezzotti; Martin Pfeffer; Anastasia Pharris; Mathieu Picardeau; Antonio Piralla; Tarja M Pitkanen; Johann David Daniel Pitout; Diamantis Plachouras; Laurent Poirel; Piero Poletti; Zvonimir Poljak; Katarina Prosenc; Milo Puhan; Joan Puig-Barbera; Caroline Quach; Mario Ramirez; Moa Rehn; Anders Rhod Larsen; Flavia Riccardo; Thomas V. Riley; Eve Robinson; Joacim Rocklöv; Ana Rodrigues; Caspar Rokx; Magdalena Rosinska; Francesca Rovida; Wallis Rudnick; Maja Rupnik; Werner Ruppitsch; Kati Räisänen; Eszter Róka; David Safronetz; Dunja Said; Jonathan M. Samet; Örjan Samuelsen; Sabine Santibanez; Milena Santric-Milicevic; Davide Sassera; Paul Saunderson; Manuel Schibler; Susanne Schjørring; Claudia Schmutz; Lena Schneider; Madlen Schranz; Eli Schwartz; Kevin Schwartz; Olivier Schwartz; Matthew Scotch; Regina Selb; Ettore Severi; Thomas Seyler; Lotta Siira; Reina Sikkema; Carolina Silva Nodari; Daniel Simoes; Lone Simonsen; Anika Singanayagam; Mairead Skally; Danuta Skowronski; Anthony Smith; David Smith; Falko Sniehotta; Gerard J.B. Sonder; Geeta Sood; Anna Maria Spagnolo; Ida Sperle; Patrizia Spigaglia; Gianfranco Spiteri; Cornelia Staehelin; Mieke Steensels; Paola Stefanelli; Pawel Stefanoff; Gyde Steffen; Lorenzo Subissi; Kathryn Suh; Vana Sypsa; Muhamed-Kheir Taha; Soroosh Tayebi Arasteh; Jakob Thannesberger; Daniel Thirion; Bruce Thorley; Jean-François Timsit; Steven Tong; Karina Top; Ana Torres; Franck Touret; Katy Town; James Trauer; Ramona Trebbien; Anne Tristan; Maria Tseroni; Gro Tunheim; Alexandra Tuttle; Soren Uldum; Wim Van Bortel; Thijs van de Laar; Miriam Van den Nest; Marc van der Valk; Sylvie van der Werf; Gary Van Domselaar; Sebastiaan van Hal; Annelies Van Rie; Jojanneke van Summeren; Jef Vanhamel; Olli Vapalahti; Marta Vargha; Tuula Vasankari; Inga Velicko; Johan Verbeeck; Pierre Verger; Elias Vermeiren; Paul Verweij; Laurence Vial; Pablo A Vial; Joaquin Vicente; Costanza Vicentini; Pilar Villalón; Jan Vinjé; Leo Visser; Chantal Vogels; Sebastian von Schreeb; Juan Carlos Vázquez-Ucha; Keita Wagatsuma; Oghenebrume Wariri; Bryna Warshawsky; Atsuyuki Watanabe; Theresa Watts; Scott Weese; Francois Xavier Weill; Nina Weis; Constanze Wendt; Dirk Werber; Maria Wessman; Henrik Westh; Therese Westrell; Heather Whitaker; Cornelia Wielders; Michelle Wille; Roel Willems; Christopher Williams; Thomas Williams; Carl-Heinz Wirsing von König; Teresa Wozniak; Benjamin G. Wu; Hsiu Wu; Claudia Wylezich; Lydia Xing; Pablo Yagupsky; Nicolas Yin; Johanna Young; Hans L Zaaijer; Cameron Zachreson; Maria Zambon; Peter Zarb; Hana Zelená; Herve Zeller; Kyra Zens; Jiawei Zhao; Yu Zhao; Ran Zhuo; Walter Zingg; Neta Zuckerman; Phillip Zucs.

